# Pregnant women, their male partners and health care providers’ perceptions of HIV self-testing in Kampala, Uganda: Implications for integration in prevention of mother-to-child transmission programs and scale-up

**DOI:** 10.1371/journal.pone.0253616

**Published:** 2021-06-29

**Authors:** Joseph Rujumba, Jaco Homsy, Femke Bannink Mbazzi, Zikulah Namukwaya, Alexander Amone, Gordon Rukundo, Elly Katabira, Josaphat Byamugisha, Mary Glenn Fowler, Rachel L. King

**Affiliations:** 1 Department of Pediatrics and Child Health, College of Health Sciences, Makerere University, Kampala, Uganda; 2 Institute for Global Health Sciences, University of California San Francisco, San Francisco, CA, United States of America; 3 Faculty of Psychology and Educational Sciences, Ghent University, Ghent, Belgium; 4 The MRC/UVRI & LSHTM Uganda Research Unit, Entebbe, Uganda; 5 Infectious Diseases Institutes, Makerere University Kampala, Kampala, Uganda; 6 Makerere University -Johns Hopkins University (MU-JHU) Research Collaboration, Kampala, Uganda; 7 Department of Medicine & College of Health Sciences, Makerere University School of Medicine, Kampala, Uganda; 8 Department of Obstetrics and Gynecology, Makerere University School of Medicine, Kampala, Uganda; 9 Department of Medicine, Johns Hopkins University, Baltimore, MD, United States of America; University of the Witwatersrand, SOUTH AFRICA

## Abstract

**Background:**

HIV status awareness is critical for HIV prevention and care but HIV testing rates remain low in Uganda, especially among men. One suggested approach to increase access and utilisation of HIV testing services is HIV self-testing. We explored perceptions of pregnant and lactating women and their male partners who attended antenatal care, and health care providers in a government hospital in Kampala, Uganda, about HIV self-testing for initial or repeat testing for women and their partners during pregnancy and postpartum We draw implications for scaling-up this new testing approach in Uganda.

**Methods:**

This was a qualitative study conducted at Mulago National Referral Hospital, Kampala, Uganda, between April and December 2017. We conducted in-depth interviews with five pregnant or lactating women and their five male partners; five focus group discussions (two with women, two with health workers and one with male partners of women attending antenatal care) and five key informant interviews with health workers providing prevention of mother-to-child HIV transmission (PMTCT) services. Data were analysed using content thematic approach.

**Results:**

There was limited awareness about HIV self-testing especially among pregnant or lactating women and their male partners. Study participants mentioned that HIV self-testing would enable people to know their HIV status faster, they thought the approach would be cost- and time-saving compared to health facility-based HIV testing, improve confidentiality and reduce stigma for those who test HIV positive. They expressed however, a general fear that HIV self-testing would lead to harm to self and others in case one tested HIV positive, including suicide, violence among couples, intentional transmission of HIV, and limited linkage to care due to lack of counselling. The likely misinterpretation of HIV test results especially among those with no or limited education, and possible coercion exerted by male partners on their wives were other potential concerns raised about the use of HIV self-testing.

**Conclusions:**

There was limited knowledge about HIV self-testing among pregnant and lactating women, their partners and health workers. While the self-testing modality was perceived to be critical for helping people, especially those in casual and distant relationships, to know their HIV status and that of their partners, most study participants believed that HIV self-testing could potentially result in a multitude of negative outcomes in the absence of pre- and post-test counselling. Successful scale-up and integration of self-testing in HIV programs requires community education, provision of information materials and making self-test kits accessible and affordable, especially in rural areas.

## Introduction

HIV status awareness is critical for HIV prevention and care but HIV testing rates remain low in Uganda particularly among men [1]. The World Health Organisation [2] defines HIV self-testing [3] as an additional approach to facility-based HIV testing [4], in which individuals collect their own specimen, perform the test and interpret the results and recommends its use in order to improve HIV testing rates. HIVST is an emerging approach with potential for high impact on HIV prevention, care and treatment [5]. HIVST has been recognized for its potential to overcome traditional barriers to testing. In particular, HIVST has been documented to be convenient and less costly [6], feasible, accurate and acceptable among the general population [7–9] including pregnant women and their partners [10–13]. Nevertheless, the risks of HIVST as perceived by pregnant and lactating women and their male partners as potential recipients of HIVST as well as their health care providers could limit acceptability and scale-up of this new testing approach. A possibility for coercive testing, inability to test accurately and difficulty to cope with reactive results in the absence of HIV counselling are some of the key concerns documented about the HIV self-testing modality [14–17]. The cost of HIVST kits and challenges regarding linkage to care for those who test HIV-positive are other obstacles [11, 12, 15–17].

In Uganda, HIVST is still a new strategy and has not yet been implemented to scale. The 2018 National Consolidated Guidelines for the Prevention and Treatment of HIV and AIDS in Uganda recommends the use of HIV self-testing as an additional approach to HIV testing services [18]. It further recommends that individuals whose HIV self-test results are positive should go for further HIV testing for diagnosis confirmation and counseling at the nearest health facility and if confirmed HIV positive, they should be linked to HIV treatment services [18]. The majority of pregnant women in many African settings including Uganda, who test for HIV as part of PMTCT programs are HIV- negative, but remain at risk of HIV acquisition during pregnancy and breastfeeding [19–21]. Acquiring HIV during pregnancy or breastfeeding increases the risks of adverse health outcomes for mothers and exposes their unborn or breastfeeding babies to higher risks of HIV infection [20, 22]. Male partner HIV testing as part of antenatal care remains low. Scaling-up HIVST could thus offer both pregnant and non-pregnant women and their male partners an opportunity for initial and repeat HIV testing and those with HIV-positive self-test results can proceed to health facilities for HIV diagnostic testing. However, successful implementation of HIVST will depend on its acceptability by intervention recipients and providers.

This qualitative study was conducted consecutive to the ‘Primary HIV Prevention among Pregnant and Lactating Ugandan Women’ [23], a randomised controlled trial (RCT) conducted between 2012 and 2017 that assessed the effect of repeat HIV testing and enhanced counselling for the primary prevention of HIV acquisition in pregnant and lactating women and their male partners during late pregnancy and throughout the breastfeeding period [24]. A qualitative evaluation of the PRIMAL intervention indicated that repeat HIV testing and enhanced counselling at health facilities were acceptable and could enhance risk reduction among pregnant and lactating women [23]. Here, we explored perceptions of pregnant and lactating women, their male partners and health care providers regarding both initial and repeat HIV self-testing for women and their male partners during pregnancy and lactation in Kampala and generated suggestions for potential integration and scale-up of HIV self-testing in PMTCT programs.

## Methodology

### Study design and population

This was a cross-sectional qualitative study conducted at Mulago National Referral Hospital in Kampala, Uganda, between April and December 2017. The study was consecutive to the PRIMAL RCT, whose methodology and quantitative outcomes have been reported previously [23, 24]. This follow-on qualitative study was conducted among selected 12 men, 22 women and 23 health care providers who either had participated in the PRIMAL study or were seeking or providing antenatal care services at Mulago Hospital (distinct numbers in each group provided in data collection section below). All study participants were from Kampala and Wakiso Districts (both of which encompass together the city of Kampala). All health workers including those who had worked in the PRIMAL study, had been involved in the national PMTCT program for over five years.

### Study setting

Mulago National Referral Hospital, located in Kampala, Uganda’s capital city, is the teaching hospital for Makerere University College of Health Sciences and other medical training institutions. HIV counseling and testing services are provided routinely to all women attending their first ANC visit daily Monday through Friday as well as to all women considered at risk of intermittent infection when presenting for delivery at maternity. Pregnant women are encouraged by the hospital staff to attend ANC with their male partners and test for HIV together. On average, 90 pregnant women are seen per ANC day.

### Data collection

Data were collected from a total of 57 study participants through 10 in-depth interviews, five focus group discussions and five key informant interviews. The 10 in-depth interviews were conducted with five lactating women, four men who took part in the PRIMAL study, and one male partner of a pregnant woman enrolled in the general PMTCT program who preferred to be interviewed as an individual instead of being part of the FGD. Female study participants from the PRIMAL study were called and selected based on their availability and interest in participating in the study. The fifth male partner who took part in an in-depth interview was recruited among men who had accompanied their women to attend ANC at Mulago but had not taken part in the PRIMAL study. We explained the purpose of this study and asked the individuals mentioned above if they were willing to participate. All accepted were invited for an interview on a day and time convenient to them at Makerere University-Johns Hopkins University Research Collaboration (MUJHU Care) which is situated on Mulago Hospital complex. Separate interviews were conducted for women and their partners.

Five focus group discussions (FGDs) of 7–10 participants each (total 42 participants) were conducted. Each FGD included a distinct group of participants: group 1) seven pregnant HIV-negative women attending ANC, group 2) 10 HIV positive pregnant women attending ANC, group 3) seven male partners of women attending ANC, group 4) 10 health workers involved in the provision of PMTCT services, and group 5) eight health workers who implemented the PRIMAL Study. The FGD participants in groups 1–4 were not part of the PRIMAL study while group 5 has been part of PRIMAL and this combination of participants facilitated comparison of perspectives regarding HIVST. Women and male partner FGD participants attending ANC were identified by attending health workers during ANC clinic visits and directed to members of the study team who provided information, recruited participants in the study and conducted the FGDs in a separate room at the hospital. Discussions were conducted by two qualitative researchers, one as a facilitator and the other as a note taker.

In addition, four health workers (a doctor, midwife, counselor and male peer counselor) involved in the provision of PMTCT services and one counselor who took part in the PRIMAL Study participated in key informant interviews.

The interview and FGD guides (included as Supporting Information) were developed by the study team informed by an extensive literature search. They explored awareness about HIV self-testing, perceived benefits and challenges of using self-testing and more specifically to reach male partners, and implications for integrating self-testing in the PMTCT program as well as the potential for scaling-up the approach. After answering the question on awareness, use of an oral HIVST kit was explained to study participants to enable them appraise its benefits and challenges. Interviews and FGDs with women and their male partners were conducted in Luganda, the main language spoken in Kampala, while those with health workers were conducted in English. All interviews and discussions were audio recorded and transcribed. On average interviews lasted 45–60 minutes and FGDs 60–90 minutes.

Data collection was phased, starting with in-depth interviews, followed by FGDs and finally key informant interviews. This approach to data collection enabled researchers to probe insights from one method of data collection to inform the subsequent data collection exercise. At the end of the interviews or FGDs, no new insights were emerging and no further data collection was undertaken.

### Data management and analysis

Data were transcribed and translated verbatim by a professional transcriber proficient in both Luganda and English. The first author (JR), who conducted all interviews and FGDs checked the transcripts for completeness. Data analysis was a continuous and iterative process guided by qualitative content thematic approach [25]. A code book was developed by the first and last author (RK) following reading of scripts several times to identify latent and manifest themes and sub-themes. Coding was done by JR who met regularly with RK to discuss emerging issues. Results were synthesised based on study themes and discussed at a study team meeting. Direct quotations were selected and used in presentation of study results. Throughout the process of data analysis, views of pregnant and lactating women, their male partners and health care providers were triangulated.

### Ethical approval and informed consent

The study was approved by Makerere University School of Medicine Research and Ethics Committee (SOMREC- Ref No. 2012–157), Uganda National Council for Science and Technology (UNCST- HS 1269), and the Committee on Human Research of the University of California San Francisco, USA (Study # 11–08151). On the day of the interview or FGD, each potential study participant was taken through the objectives, methods, procedures, benefits and risks of participating in the study and assured that no consequence was to result from their decision to participate or not. After verifying their understanding, those confirming their willingness to participate were asked to provide written consent before taking part in the study.

## Results

All women and men had more than one child, most were married or cohabiting and had completed primary education. The age range of study participants was 24–40 years.

Perceptions of women, men and health care providers regarding HIV self-testing are arranged below according to four major themes 1) awareness of HIV self-testing, 2) anticipated benefits of HIV self-testing 3) perceived disadvantages of HIVST and 4) suggestions to consider for integration and scaling up of self-testing in HIV programming **(Table 1)**.

**Table 1:** Thematic presentation of women’s, men’s and health worker’s perceptions of HIV self-testing (n = 57); Kampala, Uganda.

Major theme	Sub-theme	Women = 22	Men n = 12	Health Workers n = 23
Awareness and understanding about HIV self-testing	• Limited awareness about HIVST	☒*	☒	
	• Mistrust of test kits and results	☒	☒	☒
Anticipated benefits of HIV self-testing	• Enables people to know their HIV status faster	☒	☒	☒
	• Saves money and time	☒	☒	☒
	• Guarantees privacy	☒	☒	☒
	• Can promote faithfulness among couples	☒	☒	
	• Potential gateway to male partner testing	☒	☒	
Perceived disadvantages of HIV self-testing	• Difficulties in conducting the test and interpreting results accurately	☒	☒	☒
	• Difficulties in coping with HIV positive test results—harm to self and others	☒	☒	☒
	• Possibility of coercive HIV testing by partners	☒	☒	☒
	• Challenges to enroll in care	☒		☒
	• Challenges in disclosing HIV positive test result to partner	☒	☒	☒
Suggestions for integration and scale-up of self-testing in HIV programs	• Community education about HIVST	☒	☒	☒
	• Supportive information, education and communication materials	☒		☒
	• Make test kits accessible at no or low cost	☒	☒	☒
	• Train health workers and community leaders to promote HIVST			☒

* A checked box indicates the existence of the perceptions mentioned under sub-themes.

No box indicates the absence of the perception.

### Awareness about HIV self-testing

There was limited awareness on HIVST among pregnant women, their partners as well as health workers involved in HIV care and research. Some participants associated HIVST to other individual self-testing strategies such as the rapid pregnancy and malaria tests.

*I have not heard about HIV self-testing… maybe it is like malaria, you can buy a kit from the pharmacy and you test; but for HIV I do not know **(KII 4, Female participant PRIMAL Study)***

*What I know, it is a kit on which you put a drop of blood and…, in case it shows two lines that means you are infected but if it shows one line then you are not infected **(FGD3, Male partners of women attending ANC).***

Similar perspectives were mentioned by health workers:

*What I have heard about self-testing*, *someone goes through the process of HIV testing himself/herself without a health worker*. *She/he buys a testing kit and uses*… *drops of blood and reads the result himself or herself*… ***(*FGD 4, HWS PRIMAL Study).**

The few study participants who were aware about HIV self-testing, had accessed information mainly through friends and family members reflecting the dominance of informal networks as a source of health information.

*I heard it from my husband, he bought one testing kit and used it from home and after he said: “I know you can’t trust these results, you may think that I have faked them”…in actual sense I never trusted those results even though he works with health workers **(FGD 1, HIV negative Pregnant Women).***

*I heard two youth who were discussing… that self-testing is so easy. One said ‘I cannot have sex with any girl before testing her’…he moves with his HIV test kit. I heard them talk about it but I have never seen it **(FGD 3, Male partners of women attending ANC).***

Similar views on the importance of friends as a source of information on HIV self-testing was also evident in narratives of health workers who reported they heard friends talking about the advantage of the self-test kits.

*A friend told me that we can buy those HIV self-testing kits and use them from home; adding that we don’t need to go to the hospital to test from there since that will be time wasting* ***(*FGD 5, HWs providing PMTCT and ANC services).**

### Mistrust of HIV self-testing results

The few study participants who had heard about HIV self-testing or were encouraged to use HIV self-tests in general expressed mistrust about the effectiveness of HIVST;

*I heard that it [HIV self-testing] helps to do HIV testing but I have never seen it… One of my friends whom I told we should go for an HIV test*, *said that he had some strips that we can use*. *But I told him that I didn’t trust them*… ***(*FGD 1, HIV negative Pregnant Women).**

*My husband brought the kit home and tested himself but I never believed him because I know HIV testing should be done by trained health workers*… ***(*FGD 1, HIV negative Pregnant Women).**

Emerging from the above narratives is the fact that doubt about the effectiveness of HIVST was in part driven by lack of exposure and experience with self-testing and a belief that HIV testing should always be conducted by trained health workers.

The interviewer ensured that participants demonstrated an understanding of HIV self-testing before continuing with the interview or discussion regarding the potential uses of HIV self-testing.

### Divergent perceptions about HIV self-testing

Study participants expressed divergent and often contradictory perceptions about the likely role of HIV self-testing in supplementing the existing HIV testing approaches. In all interviews and discussions, most study participants spontaneously mentioned the likely limitations or disadvantages of HIVST. Some study participants highlighted the potential advantages of the HIV self-testing approach but still most of them argued that the new approach would have multiple disadvantages as presented below.

### Perceived advantages of HIV self-testing

The major perceived advantages of HIV self-testing were: enabling people to know their HIV status quickly, saving cost, being convenient and guaranteeing privacy.

#### HIV self-testing can enable people to know their status quickly.

Most study participants mentioned that HIV self-testing would quicken the testing process and awareness of HIV status. This could reduce the spread of HIV especially among new and casual sexual partners as study participants explained.

*It (HIV self-testing) makes it faster for one to know his/her results at any time or to know your family’s status at your convenience **(KI 1- Male partner, ANC)***

Health workers added to the same theme:

*Say for example if someone got a girlfriend outside marriage and has those kits and before going for sex they test and in case they find one HIV positive, they will not go ahead with it (sex). HIV self-testing will help to protect people from getting infected **(FGD 5, Health workers providing ANC and PMTCT services).***

*I think it (HIV self- testing) will help to reduce the number of new HIV infections*. *For example*, *if this is a new partner and they are going to have sex there and then in ‘a one-night stand’*… *one of the key issues that we want to know at that time is their sero-status*. *So if they have the kits it’s easier for them to test each other before going into the act other than going to the hospital which may be very far or closed* ***(KII 03- Health Worker Providing ANC*).**

All study participants argued that HIV self-testing would save time for traveling to and from health facilities as well as waiting for facility-based HIV testing, especially in rural areas where health facilities are far and staff is limited:

*Some health facilities are very far especially in rural areas and one can spend the whole day traveling to and from the health facility for HIV testing. In this case, self-testing can help to save time… **(FGD 1, HIV negative Pregnant Women attending ANC).***

*HIV self-testing is a good idea because it is easy to use, it saves time that would have been spent lining up at the health facility **(FGD 3, Male partners of women in attending ANC).***

The time saving advantage of HIV self-testing was also mentioned by all health workers in individual interviews and focus group discussions.

*You do not need a number to line up, you do not spend a whole day in the hospital, it (HIV-ST) saves time… **(FGD 4, HWS PRIMAL study).***

HIV self-testing was also seen as a strategy that has potential to help people in new relationships, those intending to get married or those in casual relationships who can test as couples before engaging in sex.

### HIV self-testing—a cost saving strategy

Study participants mentioned that HIV self-testing would save money that would be spent on transport to and from health facilities for HIV testing. Study participants pointed out that the amount of money saved would even be more for couples seeking to test.

*It (HIVST) minimizes costs in terms of transporting yourselves to a testing facility and it helps when it comes to the issue of time since men are always in a hurry… **(KII 1, Male Partner PRIMAL study)***

### HIV self-testing is convenient and provides an opportunity for men to test

Some study participants argued that HIV self-testing can be done any time thus making it a convenient approach unlike health facility-based testing which is subject to opening and closing hours. Self-testing was particularly considered more advantageous than health facility-based testing for people with limited time, mostly men who are often busy looking for money to take care of their families. Most study participants viewed HIV self-testing as a potential gateway to increase testing rates for male partners who in general are hesitant to attend antenatal care clinics and test for HIV with their female partners. Most participants contended that HIV self-testing can be a solution for male partners who keep giving excuses that they do not have time to travel to the health facility.

*There will be no way […] the other partner will dodge because the testing kit is right there in your house and you can’t dodge [i.e. pretend] that you do not have time… **(KI 3, Male partner PRIMAL study).***

Some male study participants viewed HIV self-testing as an opportunity for men to test themselves first before going for couple testing if they are uncertain about their HIV status. By doing so, HIV self-testing has potential to increase HIV status awareness especially among men who in general do not frequent often health facilities.

*Women have the condition of testing during pregnancy and they actually test. Self-testing will help men because most of them want their wives to test and they say that their status is the same as their wives… **(KII 3- Male partner PRIMAL).***

The potential advantage of HIV self-testing helping to test men was also mentioned by health workers:

*It [HIVST] can help people test themselves because we are still looking for avenues of bringing men on board. Self-testing is an opportunity for them to test **(KII 5, Counselor)***

### HIVST guarantees transparency and confidentiality

Most women, men and health workers alike in our study reasoned that HIV self-testing can ensure confidentiality of the result since it can be done individually, one can decide who to share with and in a private setting.

*It (HIVST) will make you know yourselves within since you are testing together; it will not expose you to other people; you will know your status among yourselves… **(KI 1, Male partner PRIMAL study)***

*You can get to know your status early without anyone else knowing; you can do it yourself and keep it to yourself **(KI 2- Male partner PRIMAL Study)**.*

One participant also explained that HIV self-testing would help to overcome likely connivance between some health workers and those seeking HIV testing to falsify ‘test results’.

*Some men say when they ask their wives to go for HIV testing, women say husbands planned with doctors to say they are negative… (**FGD, Male partners of women attending ANC**)*.

### Self-testing may promote faithfulness among couples

Study participants noted that if made available, HIV self-testing can promote couple testing which could promote faithfulness and responsibility among couples, especially those who test HIV negative and are living apart.

*I have a colleague who is a medical officer and her husband works upcountry but every month they test themselves together*. *That helps because this man will stay safe because he knows the check is on*. *Such couples can benefit from HIV self-testing kits* ***(*FGD4, HWs PRIMAL study).**

*It can help families where one of the partners is a long distance trader or works far from home*. *When he or she comes you say “Let us first test*.*” Because I do not know how you have been there and you do not know how I have been here* **(KII 4, woman PRIMAL study).**

### Opportunity to confirm test results

Some health workers in our study noted that in instances where one is in doubt of test results, self-testing can be an option to confirm HIV status.

*Some people also do self-testing to confirm the results. Like one couple that came and tested together here and turned positive; they first denied and went ahead to do it on their own to confirm **(FGD 5, Health workers providing ANC and PMTCT services).***

### Perceived disadvantages of HIV self-testing

Most study participants emphasized the potential disadvantages of HIV self-testing likely to limit its use and integration in HIV programs. The key perceived disadvantages were 1) inability to conduct the test and interpret results accurately; 2) inability to cope with positive test results in the absence of professional counseling; 3) intentional transmission of HIV to partner; 4) failure to enroll in care; and 5) the possibility of coercive HIV testing of women by their male partners.

### Inability to conduct the test and interpret results accurately

All study participants mentioned that it would be difficult to use HIV self-testing kits properly, since many people lack knowledge about the kits. Most study participants also expressed fear that test results would not be interpreted properly, especially by people with low education and rural dwellers.

*Because people do not know how to use the HIV test kits… You see like pregnancy test kits, very few people know how to use them. So, HIV self-testing will not work. Maybe here in towns or cities but in the villages it can’t work… **(KII 4, woman PRIMAL study)***

*The other problem would be that someone might test himself and gives himself negative result yet he is positive*. *This strategy is good for someone who knows how to conduct the test very well but for many of our people it will be difficult* ***(*KI1, 3 Male peer Educator)**

Similarly, health workers also expressed fear that it would be difficult for many people to conduct HIVST mainly due to low levels of education and lack of awareness about the testing approach, including test kit storage, the algorithm and interpretation.

*I do not know how we can do that because…if some people do not know how to write their names and their dates of birth, how will they interpret the strip results? That individual needs to be tested by a health worker and referred for other services **(KII 2, Health Worker)***

Emerging from the above narratives are concerns about the inadequacy of knowledge and skills about HIV self-testing which could lead to inaccurate interpretation of test results. As a results, there was a general perception that HIV testing should be conducted by health workers.

### Inability to cope with HIV positive test results

Study participants feared that HIV self-testing could lead to harm for the individual involved and others especially if test results are positive. There was widespread fear among study participants that it would be difficult for individuals to manage HIV positive results in the absence of pre- and post-test counselling by qualified health workers. All men, women and health workers in the study believed that the inability to cope with HIV positive results could lead to negative outcomes for the individual and his or her family members.

At individual level, several study participants, both men and women, feared that HIV self-testing might increase cases of suicide due to lack of counselling.

*The issue of finding yourself HIV positive where there are no counsellors—one might buy a rope and commit suicide. But if you are at a clinic in front of a health worker, he/she might tell you “this is not the end of your life, you can still live for a long time…” but if you do it alone, you might buy poison and kill yourself. **(KII 2- Male Partner).***

Some study participants argued that HIV-positive results obtained through self-testing without professional counselling could lead to worries and depression.

At the interpersonal level, there was fear among study participants that HIV-positive results obtained through self-testing without professional help from health workers could increase cases of gender-based violence within couples as one member of a couple can accuse the other of infidelity.

*In case a man tests negative and then goes ahead to test his family members and find that his partner is positive… they can hurt each other. A man can end up killing his partner. It might cause harm between them…. It is not proper to leave people at home to do self-testing without counsellors, it might cause harm in the family. **(KII 3, Male peer Educator).***

Men and women study participants noted that HIV positive test results following self-testing can lead to separation and breakdown in families.

*It may also lead to separation of couples because at that time there is no counsellor, because those people before testing they need to be counselled but now if you go straight to testing and I find my wife with the virus and yet I am safe then that woman will not sleep in my house on that day… Even the woman might decide to go after knowing that the man is HIV positive. **(KII 1, Male partner PRIMAL study).***

*You might test from home and God forbid the husband is HIV negative yet the wife is HIV positive…that will be the end of that relationship… **(KII 1, Women PRIMAL study)***

### Intentional transmission of HIV to partner

Some study participants also noted that if individuals test on their own, they may transmit HIV intentionally to their partners for fear of being abandoned or being accused of bringing the infection in the family. They could also intentionally mis-interpret the results.

*If this person tests himself or herself and gets to know that he/she is HIV positive;… he/she will say, I have also been infected why not infect other people as well! Why should I die alone?… **(FGD women attending PMTCT).***

*When you already know that you are infected and you don’t want to miss out on that person you have got, you can alter the results. It is easy to lie to someone if she does not know how to use the test kit, whatever you tell her, she might think it is correct **(FGD male partners of women attending ANC).***

### Failure to enroll in HIV care

A few study participants expressed concern that those who test HIV positive might find it difficult to enroll in HIV care due to lack of counseling, active linkage to care and not knowing where to go:

*Will people understand the way forward after testing HIV positive? My husband has tested positive and I am negative… then what next? Because these people will not be counselled before testing, they will prick and wait for the results but what will happen after the results? They will not go to the hospital for management… **(KII 1, Health worker)***

*In case he tested himself he will not encourage himself to go to the clinic and start taking ARVs. Some people will not even know which hospital to go to for treatment. Some fear going to hospitals near their homes because they do not want to be known **(KII 2- Male Partner)***.

### Challenges in disclosing HIV positive test results

It was noted that disclosure would be a challenge without health worker counselling especially to sexual partners for fear of gender-based violence or abandonment.

*Most people will not tell others when they test and find they are HIV positive. They fear to be blamed for being the ones who brought the infection in the family. It takes a lot of encouragement and support from the health worker for disclosure to happen. But this will be missing in self-testing **(FGD women attending PMTCT).***

Other challenges likely to limit the use of HIV self-testing were high cost of test kits and their limited availability especially in rural areas without pharmacies. Overall, most study participants were against promoting HIV self-testing especially due to the absence of pre and post-test HIV counseling and guidance to mitigate potential negative consequences of positive HIV test results.

### Suggestions by study participants for integration of HIV self-testing in HIV programs and scale-up

As shown in Table 1, study participants suggested 1) community education about HIVST including use of community demonstration on HIV self-testing 2) provision of supportive information, education and communication materials in local languages and sensitization on the use of HIV self-test kits to guide community members, 3) make test kits accessible at no or low cost especially in rural areas without pharmacies and 4) training of health workers and community leaders on the benefits and use of HIV self-test kits to enable them to promote the testing approach.

## Discussion

In this qualitative study, we describe perceptions of pregnant women, their male partners and health care providers regarding HIV self-testing in Kampala, Uganda and participants’ suggestions to facilitate potential integration and scaling-up of self-testing as part of the national PMTCT program.

Study findings revealed limited awareness about HIV self-testing among women, their male partners and health workers. This finding is not surprising given that this testing approach is still new and has only been part of pilot studies in Uganda [26]. The limited awareness on HIVST in this study reflects a need for raising awareness as part of scaling-up the HIV self-testing strategy. Most women and their male partners as well as health workers, expressed doubts about people’s ability to use HIV self-testing kits appropriately and to interpret results correctly, especially among people with no or low education. Taken together, the limited awareness and widespread reservations about HIV self-testing imply that both health workers and community members sustained programs to educate potential users about HIV self-testing as part of scaling-up HIV testing in Uganda and similar low-income settings.

Our study revealed divergent and often contested perceptions about HIV self-testing in supplementing the existing HIV testing strategies. Regarding the perceived benefits of HIV self-testing, most study participants mentioned that the testing approach can enable people to know their HIV status faster compared to health-facility based approaches and is cost-saving in terms of transport or time to go and wait at health facilities or testing centres. In this regard, HIV self-testing was considered suitable and with potential to reach men currently underserved by, or unwilling to test at, health facilities especially as part of maternal and child health programs [27] which largely target women. Some studies have indicated that due to difficulties of men accessing HIV testing services, some of them rely on results of their female partners and assume similar HIV status [28]. In this regard, HIV self-testing could be a gateway for men and other groups of people constrained by time to know their HIV status, and for those found to be HIV-positive to enroll in care.

In line with our findings, an earlier study conducted in Kenya, Malawi and South Africa on attributes of an ideal HIV self-test, revealed that self-testing would save on time spent waiting in long queues at health facilities and traveling to and from health facilities [17, 29, 30]. The time and cost saving benefits of HIV self-testing are of particular relevance to Uganda and other low-income settings given that COVID-19 and related restrictions have increased the cost of seeking health care. A recent qualitative study conducted in South Western Uganda revealed that COVID-19 is inhibiting access to and uptake of HIV testing services through lack of transportation, poverty and stigma related to the pandemic [31]. Fear of being exposed to COVID -19 at health facilities has also been noted [32]. Thus, prompt scale-up of HIV self–testing as part of existing HIV programs has potential to sustain access to HIV testing services in Uganda and other sub-Saharan African countries during the COVID pandemic.

The perception that HIV self-testing was convenient and guarantees privacy was recurrent in most interviews and discussions in our study. This desire for privacy among our study participants could be in part a reflection of continued stigma and the need for people to have autonomy over HIV testing and results. Being convenient, providing for privacy and autonomy as desired attributes of HIV self-testing have also been highlighted as key benefits of the testing approach by the World Health Organisation [4, 33]. Our findings on the benefits of HIV self-testing are also consistent with what has been documented in other African settings. In Ethiopia, HIV self-testing was preferred among health workers due to confidentiality [34]. A community study done in Rakai, Uganda, also highlighted privacy, convenience and ability to test before sex as key benefits of HIV self-testing [35]. Recent reviews on HIV testing approaches [17] and HIVST [30] in sub-Saharan Africa revealed that HIVST can leverage social and sexual networks to reach those unlikely to be reached by HIV programs, increases opportunities for people to test when they want, can increase testing for men, youth and key populations [17], and provides men with an alternative, confidential and convenient testing model [30]. These widely appreciated HIV self-testing benefits by women, men and health workers in our study should be building blocks for community education and the scale-up of HIV self-testing as an additional testing approach.

A unique benefit of HIV self-testing highlighted in our study is the potential to promote faithfulness among couples especially those living apart from each other including truck drivers and those in new relationships. Many study participants noted that these could undertake HIV testing as couples before engaging in or resuming sexual activity. As such, self-testing would challenge and encourage couples to be faithful to each other to avoid HIV infection. HIV self-testing has previously been found to be acceptable and feasible among truck drivers but stressed the need to provide supervised support and an opportunity for users to ask questions [36]. Our study findings also indicated that HIV self-testing can provide an opportunity for people in doubt of their HIV results to confirm their HIV status. The idea of some people doubting HIV test results given their own risk perception has also been documented in eastern Uganda [27] and can hinder HIV prevention for those who test HIV negative or enrolling in care for those who test HIV positive. It is important that, as part of activities to scale-up HIVST, community education emphasizes the need for individuals whose HIV self-test results are positive to go for further HIV testing and counseling at health facilities [18]. HIVST was also viewed to have potential to enable women and men to conduct repeat HIV-testing which is critical to enhance primary HIV prevention [11, 21, 24].

### Potential disadvantages of HIV self-testing likely to hinder scale-up

The major perceived disadvantages of HIV self-testing by our study participants were; perceived inability to conduct the test and interpret results accurately, possible difficulty in coping with positive HIV test results for self and others, a possibility of coercive HIV testing of women by their male partners, and potential failure to enroll in care following HIV positive results.

Women, men and health workers in our study expressed fear that many people would be unable to conduct HIV self-testing and interpret results accurately owing to lack of knowledge and skills. Most study participants considered HIV testing highly technical and preferred that it should be left to trained health workers. Inability to conduct HIV self-testing accurately and difficulties to cope with positive test results in the absence of HIV counselling to prepare an individual for the test and managing test results have been documented by others as barriers to scaling-up HIV self-testing [14, 16, 37]. Study participants noted that the difficulties to conduct HIV self-testing and interpret results appropriately may be more pronounced in rural areas where educational levels are low. This worry may have considerable relevance for Uganda given that the vast majority of Ugandans (close to 80%) live in rural areas [38]. Consistent with our findings, a study done in South Africa on differentiated HIV testing approaches revealed that HIV self-testing and HIV counselling and testing can co-exist and complement each other [37].

Most of the concerns related to inability to cope with the positive HIV test results and negative outcomes (separation, depression, fear of being abandoned or accused of bringing HIV to the family, intentionally transmitting HIV, violence and suicidal ideation) were linked to the absence of professional counselling and support by health workers for HIVST. These findings are not surprising given that since the advent of HIV in Uganda in the 1980s, pre and post-test counseling by health workers have been the norm and a backbone of HIV testing. These findings re-echo a need to ensure that HIV counseling is available and should be an integral part of scaling-up HIVST. Similar to our findings, a study conducted in other central Ugandan districts also documented fears of marital disruption and suicidal ideation and linked these to the absence of HIV counseling [39].

The risk of suicide following HIVST in the absence of counseling has also been documented in a qualitative study done in Kenya, Malawi and South Africa [40]. In another Kenyan study, two cases of intimate partner violence were reported by women delivering HIVST kits to their partners [41]. On the contrary, a community-based study promoting HIVST conducted in urban Malawi reported no cases of suicide or intimate partner violence attributed to HIVST [42]. Similarly, no serious adverse events were reported post-HIVST in Uganda [39], an indication that these fears may be overestimated in qualitative studies. Coercion as a social harm was reported by 3% of the respondents in the same Malawian study [42]. It is important that mechanisms for monitoring and responding to social harms following HIVST be part of plans for scale-up of the testing approach.

Other disadvantages of HIV self-testing raised in our study include limited access to test kits and their cost which may make the testing approach out of reach to low-income earners and those living in rural areas. High cost as a barrier to use and scale-up of HIVST has been highlighted in other African studies [16, 17, 43]. The concerns about affordability as a likely barrier to use of HIV self-testing are relevant given that 21.4% of Ugandans are estimated to be living below the poverty line [44]. The implication here is that making HIVST accessible and affordable will be key to its utilization.

To counter the disadvantages likely to hinder scaling-up of HIV self-testing in Uganda, study participants recommended public education about the testing approach, provision of information and education materials including those with clear instructions and visual aids, as well as training of health workers to promote HIV self-testing. The implication here is that the potential scale-up of HIV self-testing in HIV programs should go hand in hand with a strong behaviour change communication component highlighting the benefits and how to minimize the potential disadvantages of the approach.

Similar to our findings, a study on men’s experiences of HIVST in Rwanda recommended health education and awareness creation about the testing approach and ensuring that test kits are available at a low cost. Also making self-test kits accessible over the counter or in the same manner as condoms including distribution through home visits, use of call centres for ordering kits and collection from local leaders, supermarkets and pharmacies [43] are other strategies to improve access. A review of men’s perspectives on HIVST in sub-Saharan Africa recommended the use of community-level campaigns to educate men about HIVST and implementing strategies to ensure potential HIVST users are counseled and supported to test [30]. Having user friendly HIVST kit instructions [16] and translating them into local languages [43] are other key strategies to improve use of the HIVST.

Our study findings should be interpreted in light of the following strengths and limitations.

Use of qualitative methods of data collection facilitated an in-depth understanding of perceptions regarding HIV self-testing and what might be needed for its scale-up. The inclusion of pregnant and lactating women, their male partners and health care providers in the study enabled triangulation of data which helped to improve the trustworthiness of our findings. However, the study was conducted at a public hospital in the general antenatal care setting, thus the results apply to the population at this type of health facility but may not reflect the perceptions of women and men of higher socio-economic status. As well, the study was conducted at a tertiary hospital with experienced health workers often involved in research, and whose awareness about HIVST may be higher than what may be found at lower level and in rural health facilities with minimal exposure to research. The study was also conducted in Kampala, the capital city of Uganda, whose population tends to be more educated and informed. Thus the awareness and perceptions of this population may be different from those of rural dwellers. Nevertheless, the fact that our findings especially regarding the benefits and disadvantages of HIVST were similarly documented in other Ugandan settings [13] and other African countries is reassuring that our findings may have wider applicability beyond the study area.

Data for the study was collected in 2017 before Uganda started rolling out HIVST. With ongoing rollout, awareness might have slightly improved given that the rollout process has been slow. Thus more studies are needed to assess changes in awareness and stakeholder perceptions about HIVST as the testing modality becomes more available in Uganda and other African settings. In this study, we did not demonstrate how the HIVST kit works which would have improved study participants’ familiarity with the kit. Indeed, some of the study participants kept referring to use of blood samples possibly reflecting as a common approach of collecting HIV testing samples through finger pricks which they have been able to experience or witness at most health facilities in Uganda. Future studies should include a demonstration session on the use of HIVST kits. However, given that our findings on awareness, benefits and limitations of the HIVST testing approach are consistent with what has been documented in Uganda [11, 13] and other African settings [15–17, 30] supports the validity of our findings.

## Conclusion and recommendations

Although there was limited knowledge and experience about HIV self-testing among pregnant and lactating women, their male partners and health workers in Kampala, HIV self-testing was perceived as having the potential to help individuals and couples, especially those in casual and distant relationships to know their HIV status and that of their partners faster, more conveniently, saving cost and time, and guaranteeing privacy and confidentiality. The approach was also deemed to possibly encourage partners to be faithful to each other and could be an important tool for the integration of repeat postpartum testing into PMTCT programs. The major potential disadvantages of HIV self-testing perceived were a risk of harm to self or others including domestic violence and family breakdown in case of positive HIV self-test results as well as poor linkage to care due to the absence of pre and post-test counselling. Thus successful integration and scale-up of HIV self-testing in Uganda will require raising awareness and ensuring adequate linkages among health workers and community members about this new testing approach. Provision of information, education and communication materials including use of visual aids, making test kits available at no or low cost, and ensuring available and functional counselling support to users of HIVST especially in rural areas are key to scaling-up. Further research is needed to understand changes in awareness and perceptions about HIVST as the strategy is being scaled-up in Uganda and other African countries.

## Supporting information

S1 File Interview/Discussion guide for PRIMAL study on HIV self-testing in Kampala in English. (PDF)

S2 File Interview/Discussion guide for PRIMAL study on HIV self-testing in Kampala in Luganda. (PDF)

S3 File Interview excerpts by major themes and sub-themes perceptions of HIV self-testing in Kampala. (PDF)

## List of abbreviations

ANC: Antenatal Care
HWs: Health Workers
FGD: Focus Group Discussion
HCT: HIV Counselling and testing
HIVST: HIV self-testing
IDI: In-depth Interview
KII: Key Informant Interview
PMTCT: Prevention of mother-to-child transmission of HIV and WHO: World Health Organization
