# Supplementary material for: Pregnant women, their male partners and health care providers’ perceptions of HIV self-testing in Kampala, Uganda: Implications for integration in prevention of mother-to-child transmission programs and scale-up
Source: PLoS One. 2021 Jun 29;16(6):e0253616. doi: 10.1371/journal.pone.0253616 (PMC8241041; doi:10.1371/journal.pone.0253616)
Supplement: S1 File — (PDF) [file pone.0253616.s001.pdf]

INTERVIEW /DISCUSSION GUIDE FOR PRIMAL QUALITATIVE STUDY ON HIV TESTING IN KAMPALA

Study Participants (breastfeeding women and their male partners who participated in the PRIMAL study, pregnant HIV-negative women attending ANC, HIV positive pregnant women attending ANC, male partners of women attending ANC, health workers involved in the provision of PMTCT services, and health workers who implemented the PRIMAL study)

Part 1: Identification Information: Obtain and record key identification information (age, sex, category of participant (male partner, woman in ANC/PMTCT/Primal, HW (PRIMAL or PMTCT), home district, level of education, marital status, for HWs length of time involved in PMTCT).

Part II: Awareness and perceptions of HIV self-testing

1. What are some barriers women face in getting partners to test for HIV during pregnancy/lactation?

2. Have you heard about the HIV self-test? If so what have you heard about it and what were your sources of information on this?

Note: For study participants who have not heard about HIV-self testing, explain HIV self-tests using saliva (before proceeding to other questions). A process in which individuals collect their own specimen (saliva), perform the test and interpret the results. Those who test HIV positive should go to the health facility for confirmatory test and guidance on starting HIV treatment.

3. What do you think might be the advantages of using the HIV self-test by women and their male partners? (Probe for benefits of HIVST for initial and repeat testing during pregnancy and breastfeeding, if not mentioned)

4. What do you think might be the major barriers to use the HIV self-test by women and/their male partners?

5. What is your view about integrating HIV self-testing in the PMTCT program?

6. What should be done to improve use of HIV self-testing for initial and repeat HIV testing among women and their partners during pregnancy and the breastfeeding period?

7. Any other comment?

Thank you for your time and views.
