# Supplementary material for: Pregnant women, their male partners and health care providers’ perceptions of HIV self-testing in Kampala, Uganda: Implications for integration in prevention of mother-to-child transmission programs and scale-up
Source: PLoS One. 2021 Jun 29;16(6):e0253616. doi: 10.1371/journal.pone.0253616 (PMC8241041; doi:10.1371/journal.pone.0253616)
Supplement: S2 File — (PDF) [file pone.0253616.s002.pdf]

EBIBUUZO EBINAABUUZIBWA/EBINAAKUBAGANYIZIBWAKO EBIROWOOZO MU KUNOONYEREZA OKUKWATA KU KUKEBERA AKAWUKA KAAMUKENENYA MU KAMPALA

Abeetabye mu kunoonyereza (Abakyala abayonsa n'abaami baabwe abeetaba mu kunoonyereza okuyitibwa 'PRIMAL study', abakyala abali embuto nga tebalina kawuka kamukenenya abajja ku kiliniki y'abakyala abali embuto, abakyala abalina embuto nga balina akawuka kamukenenya abajja mu kiliniki y'abakyala abali embuto, abaami b'abakyala abali embuto abajja mu kiliniki y'abakyala abali embuto, abasawo abakola mu nteekateeka egenderera okuziyiza ba maama abali embuto okusiiga abaana baabwe akawuka kamukenenya n'abasawo abaakola okunoonyereza okuyitibwa 'PRIMAL study')

Ekitundu 1: Ebikwata ku beetabye mu kubuuzibwa ebibuuzo: Funa era wandiika ebintu ebikulu ebikwata ku beetabye mu kukubaganya ebirowoozo/okubuuzibwa ebibuuzo (emyaka , ekikula kyaabwe , ekiti eyeetabye mu kunoonyereza mwaagwa (mwami wa mukyala, omukyala ajja mu kiliniki y'abakyala abali embuto/omukyala ali mu nteekateeka eya PMTCT oba Primal, disitulikiti gyebava, omutendera gw'obuyigirize, oba bafumbo, ne bbanga abasawo abakola mu nteekateeka eziyiza bamaama okusiiga abaana baabwe akawuka (PMTCT) lye bamaze mu nteekateeka eno).

Ekitundu II: Okumanya ebifa ku kwekebeza akawuka kamukenenya n'ebirowoozebwa ku nteekateeka eno

1. Biki ebiremesa abakyala okuleeta abaagalwa baabwe okukeberebwa akawuka kaamukenenya mu kiseera nga bali mbuto/nga bayonsa?

2. Wali owulidde ko enkola eyomuntu kinomu kwekebera akawuka kaamukenenya? Bwekiba kituufu, biki byewawulira ku kwekebeza kuno era wabiwulira kuvaawa?

Wetegereze: Eri abo abeetabye mu kukubaganya ebirowoozo nga tebawulirangako ku kwekebera kawuka kaamukenenya, nnyonnyola okwekebera akawuka kaamukenenya nga tukozesa amalusu (nga tonabuuza bibuuzo birala). Eno y'engeri omuntu gyeyeggyako amalusu, naagakebera era n'amannya ebivudde mu kukebera kuno. Abo abasaangibwa nga balina akawuka kaamukenenya bateekeddwa okugenda mu ddwaliro okukola okwekebeza okukakassa kino n'okuweebwa amagezi ku kutandika okumira eddagala ly'akawuka kaamukenenya.

3. Olowooza birungi ki ebiyinza okubeera mu omuntu kwekebera akawuka kaamukenenya yeka? (Buza ku biringi ku bakyala n'abaagalwa baabwe mu kiseera ng'abakyala bali mbuto ne mu kiseera eky'okuyoonsa, kukebera akawuka kamukenenya omulundi ogusoka oba kuddamu kukeberebwa bwebiba tebyoggeddwa?)

4. Olowooza biki ebiyinza okulemesa abakyala n'abaagalwa baabwe okukozesa enkola ey'okwekebera akawuka kaamukeneya?

5. Kiki ky'olowooza ku kugatta enkola eno ey'okwekebera akawuka kamukenenya n'enteekateeka ey'okuziyiza bamaama okusiiga abaana baabwe akawuka kaamukenenya eyitibwa PMTCT?

6. Kiki ekirina okukolebwa okutumbula enkola ey'okwekebera akawuka kaamukenenya mu kuddamu okwekebeza akawuka kano mu bakyala n'abaagalwa baabwe mu kiseera ng'abakyala bali mbuto ne mu kiseera eky'okuyoonsa?

7. Olina/Mulina ekirala kyemugamba?

8. Mwebale nnyo olw'obudde n'ebirowoozo byamwe byemuwaddeyo
