# Supplementary material for: Pregnant women, their male partners and health care providers’ perceptions of HIV self-testing in Kampala, Uganda: Implications for integration in prevention of mother-to-child transmission programs and scale-up
Source: PLoS One. 2021 Jun 29;16(6):e0253616. doi: 10.1371/journal.pone.0253616 (PMC8241041; doi:10.1371/journal.pone.0253616)
Supplement: S3 File — (PDF) [file pone.0253616.s003.pdf]

S3 File. Interview Excerpts by major themes and sub-theme - Pregnant women, their male partners and health care providers' perceptions of HIV self-testing in Kampala, Uganda

**Major Theme**	***Sub-themes and quotes***
Heard about HIV self-testing	***Awareness about HIV Self testing***<br><br>*Have you heard about self-testing? Yes, I have. You test yourself individually when you are at home, at your place…anywhere. Have you had someone consulting you about self-testing? Yes. Okay they come and they are like "My husband is in the army and they were given kits at their workplace to come and test us." They also tell me the results…whatever results they get. So, in case of negative results, we still give them information of coming back to the hospital and test because the results they get can be affected by a number of things. Some of them don't use buffers because it is difficult for every person you give a kit or a strip to be given a buffer. So, they use water, they use normal saline and those are some of the things which might affect the results. And still, you also need some bit of knowledge to carry out that test. Otherwise I see the issues of getting false negatives could be higher (KII- 1 Health Worker).*<br><br>*I haven't heard about it but I guess you just get a strip and test yourself. I do not know whether that is the real thing used (KII- 2 HW at PMTCT)*<br><br>*I have heard of it. On world AIDS day there is a certain clinic which was telling us that they give HIV testing kits to people to test themselves (KI- 5 Counselor).*<br><br>*I have heard about it before but that was when I had joined this program (PRIMAL STUDY) and they were sensitizing health workers about it but I haven't seen it being done yet (KI 3- Male Peer*

	Educator).<br><br>*One was told by a friend that "We can buy those kits and use them from home; we don't need to go to the hospital to test from there, it is time wastage." They then went to the pharmacy, bought the kits and tested themselves (FGD 4, HWs PRIMAL Study).*<br><br>*Some wives do have spouses who are health workers, they do not accept to come to the hospital and instead get the kits and test from home (FGD 4, HWs PRIMAL Study).*<br><br>*Some have multiple partners and they keep testing themselves from home (FGD 4, HWs PRIMAL Study).*<br><br>*What I have heard about self-testing, someone goes through the process of HIV himself without a health worker. She buys a testing kit and uses it puts there little drops of blood and reads the result himself or herself. But sometimes some people collapse when they misinterpret the results. Some accept the results the way they are especially the positive cases because she or he knows the way she has been behaving. So, it is not easy, it can make someone collapse without other members who are around noticing why she has collapsed (FGD 4, HWs PRIMAL Study).*<br><br>*These days, because of the technology many people have resorted to HIV self-testing because you can access the testing kit even if you go to a pharmacy and you want a testing kit they can give it to you but they don't tell you how to use it. so, you find that someone wants to test maybe his girlfriend or husband and they come "Can you please show me how to test" and these days I get so many of them "Help me interpret these results because I have my girlfriend" especially in the elite. There is a study which showed that many people do not want to use condoms so they resorted to using HIV testing kits; as they test they do it there and then. (FGD 4, HWs PRIMAL Study).*

	I have not heard about HIV self-testing. No, maybe it is like malaria. You can buy a kit from the pharmacy and you test; but for HIV I do not know (KII 4, Female participant PRIMAL study)<br><br>*What I know, it is a kit on which you put a drop of blood and . . ., in case it shows two lines that means you are infected but if it shows one line then you are not infected (FGD3, Male partners of women attending ANC).*<br><br>*Yes. You get a kit and test yourself. I have also ever tried to test myself to know whether I was pregnant came out that I wasn't pregnant ((KII 3, Female participant PRIMAL study).*<br><br>*I heard two youth who were discussing . . . that self-testing is so easy. One said 'I cannot have sex with any girl before testing her'…he moves with his HIV test kit. I heard them talk about it but I have never seen it (FGD 3, Male partners of women attending ANC).*<br><br>*I have heard that those testing kits are being sold in pharmacies where people can test themselves but I have never done that before (KII 3, Male Partner PRIMAL Study).*<br><br>*I have ever heard about it. I heard that it (HIV self-testing) helps to do HIV testing but I have never seen it… (FGD 1, HIV Negative Pregnant Women).*<br><br>*A friend told me that we can buy those HIV self-testing kits and use them from home; adding that we don't need to go to the hospital to test from there since that will be time wasting (FGD 5, HWs providing PMTCT and ANC services).*<br><br>*That there is a kit they use now days whereby you can also test for HIV at your home. One, I have*

	never seen it, it is not popular because most people are not aware that you can test for HIV when you are at home (KII 1, Male Partner PRIMAL study).
Advantages of HIV self-testing	***Enables people to know their HIV status quickly/saves time***<br><br>*The advantages are that the client gets to know…if done well, the client gets to know their sero-status even without the help of a health worker. .. I think it (HIV self-testing) will help to reduce the number of new HIV infections. For example, if this is a new partner and they are going to have sex there and then and in 'a one- night stand' if I may use that, one of the key issues that we want to know at that time is their sero-status. So if they have the kits its easier for them to test each other before going into the act other than going to the hospital which may be very far or closed (KII 3, HW in ANC).*<br><br>*It (HIV self-testing) is easy to get your results faster (KII 2, HW ANC).*<br><br>*I also think it empowers people to do HIV testing and reduces that fear of going to the facility…it is an empowerment. (KI- 5 Counselor).*<br><br>*If you are taught and you know what to do then you can actually do it. And to also know the status of all people as a household. If you a father [household head] and you have tested yourself...because these days they encourage everyone to test in a household…it would be a good idea because if you test yourself, test the wife and the children, house helper and any other in the house. Because, as we do community visits, we are unable to reach everyone…so, I think self-testing can help (KI- 5 Counselor).*

	It (HIV self-testing) makes it faster for one to know his/her results at any time or to know your family's status at your convenience (KI 1-Male partner, ANC)<br><br>*It is good. It helps you to know your status. To know that you are not infected. I do not see anything bad with it if it is done correctly. Unless you do it in a wrong way but if it is done correctly then it can give you what you want (KII 3- Woman PRIMAL study)*<br><br>*I think if the people who are doing self-testing know exactly how to interpret what they are seeing on those strips and there it could help. Say for example if someone got a girlfriend outside marriage and has those kits and before going for sex they test and in case they find one HIV positive they will not go ahead with it (sex), HIV self-testing will help to protect people from getting infected. Maybe because it saves time (FGD 5, HWs Providing PMTCT and ANC services).*<br><br>*The first advantage is that it is easy to do and it is done right from home without having to travel long journeys and it solves issues of lack of time or being so busy. I think it might be easy. It is done from home and one is able to know by herself whether she is infected or not (FGD 1, HIV Negative pregnant women).*<br><br>*It (HIV self-testing) is cheap in terms of time. Nobody will know your results. You do not need a number to line up. You do not need a number to line up, you do not spend a whole day in the hospital, it (HIV self-test) saves time. You are able to know your status as couple (FGD 4, HWs PRIMAL study).*<br><br>*Some health facilities are very far especially in rural areas and one can spend the whole day*

	traveling to and from the health facility for HIV testing. In this case, self-testing can help to save time going to the hospital and waiting there (FGD 1, HIV negative Pregnant Women attending ANC).<br><br>*I think that makes it easier for people to test themselves because he will get to know whether is infected or not and keep it confidential (KII – 1 Women PRIMAL study)*<br><br>*HIV self-testing is a good idea because it is easy to use, it saves time that would have been spent lining up at the health facility (FGD 3, Male partners of women attending ANC).*<br><br>***Cost saving strategy (saves money and time)***<br><br>*It (HIV self-test) minimizes costs in terms of transporting yourselves to a testing facility and it helps when it comes to the issue of time since men are always in a hurry (KII-1 Male Partner Primal).*<br><br>*It reduces on the time that person would spend going to the health facility…. (KI- 5 Counselor).*<br><br>*HIV self-testing save money. You can do it at home instead of going to the health centre (FGD 3, Male partners of women attending ANC).*<br>*Instead of going to the hospital and wait for long to test for HIV you can do it at home and save the time and money for transport ((FGD 4, PRIMAL study).*

	The other good thing is that it does not require a lot of time or making lines (KII 3- Male Partner PRIMAL study).<br><br>***Guarantees transparency and confidentiality***<br><br>*It (HIV self-test) will also make you know yourselves within since you are testing together. It will not expose you to other people; you will know your status among yourselves. (KII-1 Male Partner PRIMAL Study).*<br><br>*You get to know your HIV status. It is confidential (FGD 2- Women attending PMTCT)*<br><br>*You get to know your status early without anyone else knowing; you do it yourself and keep it to yourself (KII -2 Male partner PRIMAL Study).*<br><br>*Men can decide to test themselves without their partners knowing. The other good thing is that it does not require a lot of time or making lines (KII 3- Male Partner PRIMAL study).*<br><br>*You can get to know your status early without anyone else knowing; you can do it yourself and keep it to yourself (KI 2- Male partner PRIMAL Study).*<br><br>***Convenient and provides an opportunity for men to test***<br><br>*The third one, there will be no way whereby the other partner will dodge because the testing kit is right there in your house and you can't dodge (ie pretend) that you do not have time. So men will*

	test also (KII-3 Male Partner PRIMAL study).<br><br>*Some men say when they ask their wives to go for HIV testing, women say husbands planned with doctors to say they are negative. So self-testing can help since both of them will be present (FGD 3, Male partners of women attending ANC).*<br><br>*It is good because you get to know the status of each other but it might be disadvantageous because most of us haven't gone to school and might not know how to use it (KII- 1 Male partner PRIMAL Study).*<br><br>*Women have the condition of testing during pregnancy and they actually test. Self-testing will help men because most of them want their wives to test and they say that their status is the same as their wives. So self-testing can help to reach men. (KII 3- Male partner PRIMAL).*<br><br>*It [HIVST] can help people test themselves because we are still looking for avenues of bringing men on board. Self-testing is an opportunity for them to test (KII 5, Counselor)*<br><br>***Promote faithfulness among couples and new ones to know their HIV status***<br><br>*It is good if you have separated or when someone is far and when he comes back you first go for a test and then go with him (KII- 2 Woman PRIMAL study)*<br><br>*It can help those families where one of the partners is a long distance trader or works far from home. When he or she comes you say "Let us first test." Because I do not know how you have been*

there and you do not know how I have been here (KII-04 Woman Primal Study).

It can help the new couples; those that are going to start-up a family. It can help if they know how to use it properly. Before they start a family they get to know their status so that they can stick to each other (KII-04 Woman PRIMAL Study).

...mostly the new couples; it would help them. And also as I told you, you know these men work far more than women. So you do not know, a man might come out and say "I have a kid" and you do not know the status of the other woman. So it can also help there. Even for those who might be HIV positive and they think of going to the witch doctor, maybe it can help one to test before going to the witch craft (KII 04, Woman PRIMAL study)

I have a colleague who is a medical officer and her husband works upcountry but every month they test themselves together. That helps because this man will stay safe because he knows the check is on. Such couples can benefit from HIV self-testing kits. They know that anytime I go home there will be a test done (FGD 4, HWs PRIMAL study)

HIV Self-test provides an opportunity to confirm test results

Some people also do self-testing to confirm the results. Like one couple that came and tested together here and turned positive; they first denied and went ahead to do it on their own to confirm (FGD 5, HWs providing ANC and PMTCT services).

Maybe on top of that, some men have a trick of first testing women and then go ahead to have sex (FGD 5, HWs providing ANC and PMTCT services).

In cases where people do not believe their test results from a health centre, they can test

	themselves and confirm … (FGD 4, HWs PRIMAL study)
Disadvantages of HIV self- testing	***Mistrust of self-testing process***<br><br>*I have ever heard about it. I heard that it (HIV self-testing) helps to do HIV testing but I have never seen it…It is one of my friends whom I told we should go for an HIV test said that he had some strips here that we can use. But I told him that I didn't trust them and I never saw them (FGD 1, HIV Negative Pregnant Women).*<br><br>*My husband bought the kit and tested himself but I never believed him because I know HIV testing should be done by trained health workers. After he said "I know you can't trust these results, you may think that I have faked them" and in the actual sense I never trusted those results even though he works with health workers (FGD 1, HIV Negative Preganant Women).*<br><br>*Because many people are not educated, I can't trust the results they get by testing themselves (FGD 4, HWs PRIMAL).*<br><br>*My partner might come with it and goes ahead to test me and himself but since I don't know how to interpret the results I might still not be in position to tell the correct results. I feel I do not trust that method. (FGD 1, HIV Negative Pregnant women)*<br><br>***Inability to conduct the test and interpret results accurately***

	The disadvantages of course they talked about misinterpreting the results and they always use determine and we all know that it is not easy to use it without adding on other like the guidelines show. So, it is very common these days (FGD 4, HWs PRIMAL).<br><br>*Because people do not know how to use the HIV self-test kilts, then it will be hard. You see like pregnancy test kits, very few people know how to use them. Because you can listen to an advert that it crosses two lines or one line but they will not know exactly where. It is like how they advertise condoms, some do not know how to put them on, they do not know how to use them and they do not know how to keep them safely. So, HIV self-testing will not work. Maybe here in towns or cities but in the villages it can't work. It can be confusing (KII-04, Woman PRIMAL Study).*<br><br>*…and some might test and say "I am negative" when is not. You know you can make it invalid or you can use it and it gets invalid. You can see one line and say that you are fine yet you have used it badly. And others do not know English yet the information on it is in English. Others can read but they cannot understand it, others may not even know how to read (KII -04 Woman PRIMAL Study).*<br><br>*Sometimes you might find that they have misinterpreted the results. I do not know how we can do that because…if some people do not know how to write their names and their dates of birth, how will they interpret the strip results? That individual needs to be tested by a health worker and referred for other services (KII 2, Health worker)*<br><br>*...lack of knowledge in using those kits…then the interpretation of some of those strips is hard. For example; you can get a strip and you have a cross on the patient result but then you have nothing on the control. So that is already an invalid test but someone might take it as living positive or*

	negative depending on what they have been taught. (KII- 01 Health Worker providing ANC).<br><br>*The other problem would be that someone might test himself and gives himself negative result yet he is positive. This strategy is good for someone who knows how to conduct the test very well but for many of our people it will be difficult (KII, 3 Male peer Educator)*<br><br>*The one I faced [encountered] is some time back a Muslim who had two women. He was negative and the second wife was positive. After getting the results, he left the woman and walked away. When she returned to the clinic for her visit she told me that "Hajj said that I knew I was HIV positive and I wanted to kill them."...polygamy would be part of the barriers to self-testing… (KII – 5, Counselor).*<br><br>*It might not be easy because the person testing themselves must have the knowledge of the algorithm of HIV testing. They might not do it well…even the storage of the testing kit; when you buy it how do you store it! …the temperature, heat…and also wrong interpretation of the results because that test has a control line and a result line (KII- 5 Counselor)*<br><br>*Not everyone knows how to read off the results from the strip. This means that you require a lot of time to train each and every one on how to use it well. The other problem would be that someone might test himself and gives himself negative results yet he is positive. He might also get negative results yet he is positive in case he uses something else which is not buffer. This strategy is good for someone who knows how to conduct the test very well (KI-3 Male partner PRIMAL)*<br><br>*It (self-testing) can lead to death. For example, they can test as a couple and you find that one is positive which then makes that person lose hope. Many people do not know how to use those testing kits (FGD 5- Health workers providing PMTCT and ANC)*

	They may get faulty results. This is something that determines someone's future, they may get wrong results (FGD 5- Health workers providing PMTCT and ANC).<br><br>*Some may lie their spouses that "This shows that you are negative yet it means the other way round because you do not have the knowledge; and you will just take that (FGD 4 HWs-PRIMAL study).*<br><br>*He can test himself and tell you that he is okay [HIV negative] yet he is lying to you (KII-03, Woman PRIMAL Study).*<br><br>*I want to add on what she has said. Well, I am doing self-testing but I am not a technical person in that area. I may not be well versed with the guidelines of HIV testing and this may happen. You may find that one has false results and there might be misinterpretation of the results. So I don't encourage it for the general community though I do it (FGD 4, HWs PRIMAL study).*<br><br>*False results. And most of them are done without counselling and the outcome might be bad. It requires a bit of medical knowledge before using it (self-testing). Some just get a strip and prick without the sterile liquid. When someone is still in that window period when he has just gotten infected it may not show the true results (FGD 4, HWs PRIMAL study)..*<br><br>*The people testing are not trained; most households are illiterate (KII 1, Male Partner Primal study).*<br><br>*You might actually know how to use it (HIV self-test) but does your partner also know? You might lie to her that you are not infected yet you are. I do not support that method. No I don't because we do not have same level of understanding and she might end up infecting me because I am ignorant of how it can be used. It is good for those who know how to use it well but I do not support it in*

	those who are ignorant (KII- 4 Male partner PRIMAL Study).<br><br>*I do not think it is safe because even for malaria they need the other water to pour there and the thing moves and I do not think everyone knows how to use it. It is not safe; it can be used by few people. The ones who have knowledge about it. Now like me, I didn't even know that it was there (KII- 4 Woman PRIMAL study).*<br><br>***Inability to cope with HIV positive test results***<br><br>*In case a man tests negative and then goes ahead to test his family members and find that his partner is positive and there is no counsellor to counsel them, they can hurt each other. We have heard so much violence between couples. A man can end up killing his partner. It might cause harm between them… There is need for counsellor at that moment. It might lead to separation in case one is positive and the other negative. It is not proper to leave people at home to do self-testing without counsellors, it might cause harm in the family (KI 3- Male peer educator).*<br><br>*It can also lead to violence. Where a couple tests and both are positive, that can lead to violence, unlike at the hospital where there is a counsellor to help them. It can also increase crime cases where someone is positive and decides to commit suicide (FGD 4, HWs PRIMAL study).*<br><br>*However much we empower women to do self-testing to bring their partners on board, but we do not know what is going to happen at home, if a woman is the one who is HIV positive and the man is negative, don't you think it will increase on domestic violence whereby we are going to find many women being killed because of being positive and partner negative? (FGD 4, HWs PRIMAL study).*

	If one finds out that the other is positive and he/she is negative, then he/she may leave the other because there is no counselling. If a man finds out that he has he might make sure that even the woman gets it; he can force himself on her. Or if a man finds out that a woman has he might even kill her. He will say that she has not been trustworthy how come she is infected! Families may breakup (KII 04, Woman PRIMAL study).<br><br>*...But the bad thing with it is that the issue you are dealing with is so heavy. The issue of finding yourself HIV positive where there are no counsellors one might buy a rope and commit suicide. But if you are at a clinic in front of a health worker he/she might tell you that "This is not the end of your life, you can still live for a long time" but if you do it alone, you might buy poison and kill yourself (KII- 2 Male partner PRIMAL study).*<br><br>*And no one will even provide any counselling. He might say "I always leave home to go look for money but you stay here to do prostitution." and a fight breaks out. And the other thing, you know, you might test yourselves and find out that both of you are actually infected but then that takes you a lot of time to settle down and accept what you are. Even when you are in a hospital setting it has always been challenging; still people end up falling sick and losing weight. But at least you are able to receive some counselling when you have it in a hospital. From home one may develop pressure (FGD 1, HIV negative pregnant women)*<br><br>*It may also lead to separation of couples because at that time there is no counsellor, because those people before testing they need to be counselled but now if you go straight to testing and I find my wife with the virus and yet I am safe then that woman will not sleep in my house on that day because no one has been around to counsel them that one partner might have HIV and the other may not be having. Even the woman might decide to go after knowing that the man is HIV positive (KII 1, Male Partner Primal study).*

	I think that he might go ahead to test you and test himself as well but if he finds out that you are positive and he is negative I think we might end up fighting; he might beat you up calling you a prostitute (FGD 1, HIV Negative Pregnant women)<br><br>*In case you test and find out that you are infected without receiving counselling, you might develop stress and end up dying early yet in a hospital a health worker would have counselled you to avoid that. If you are counselled by a health worker, she might advise you to start treatment and protect your life. But if you did self-testing you might decide to be confidential with your results and fail to start treatment (KII- 2 PRIMAL study).*<br><br>*You might test from home and God forbid the husband is HIV negative yet the wife is HIV positive that will be the end of the relationship. Most men are never patient if it is the woman infected he might look at you as someone who died sometime back and you just want to infect him. But if it is the other way round, a man might be in fear of being left (KII-1 Women Primal Study).*<br><br>*The other thing; there is no follow-up. There is no one to follow-up because they didn't get the opportunity of someone counselling them. It may lead to separation. Like you may test me and I am positive yet you are negative and then decide to separate yet you may not be sure of my results. You might have tested me wrongly and then we separate there and then yet I am not positive (FGD 5- Health workers providing PMTCT and ANC).*<br><br>*...we do not know even the procedure of testing yourself at least when you go there those people [health workers] were taught how to test. At least they (HWs) know better than testing from home. And you might test and find that you are HIV positive and no one to counsel you. Maybe someone may kill himself or herself when she finds out that he/she has HIV but at least in a hospital they first give that person advice, they counsel. ...if they find that a man has AIDS maybe can want to kill a woman that is the one who brought that AIDS (KII- 02- Woman PRIMAL study).*

	Even the woman might decide to go after knowing that the man is HIV positive and for her she is okay. She will say "How can I get infected when I am just looking on? I rather go away and get another man."...you know men are very fast in judging. In most cases these women; especially if she has children she might say "Let me stay around and look after my children" although she may also shy away from having sexual affairs with the man but since they are sensitized about use of a condom they might opt for that. There the value of a condom will be realized. But if it is on the side of the woman the man will try to chase her away. So these people need to be counselled first. But a woman can endure and remain (KII-01, Male Partner PRIMAL Study).<br><br>*It (self-testing) is not a good method, it involves dying. Even when you are tested from a hospital a health worker takes time to tell you and by the time she gets to tell you the mind is a bit settled. But for self-testing, the moment you put blood on the strip you immediately get the results (FGD 1, HIV Negative pregnant women)*<br><br>*It (HIV self-testing) can increase immorality and then people don't consider other issues like infections, unnecessary pregnancies, they just test for HIV and go ahead [with sex]. But after getting unwanted children they neglect the woman leading to immorality. Some may not have the knowledge of the incubation period. A person may use those kits yet they are already expired and the results they get they may think are the right results yet they are not (FGD 5, HWs providing PMTCT and ANC).*<br><br>*The other thing is, if men still blame women for infecting them after being counselled in a hospital, how about when it is just the two of you at home! I think that is real trouble because he can beat you to the extent of dying (FGD 1, HIV Negative pregnant women)*

	It would have been a good method but in case I knew I was HIV negative only to realize that I am positive and there is no counselling I might get stressed up and feel like taking poison, but when I come to a health centre there is a way health workers handle you, counsel you and leave when you have cooled down (FGD 2, Women attending PMTCT).<br><br>*I do not see any good thing out of it if we had never had an HIV test because in case it shows that you are HIV positive you might end up doing something bad to yourself since there is no ne counselling you. But if you go to a hospital health worker will know how to handle you and you leave well. In a hospital it is easy to lose energy, how about when you are at home alone! (FGD 2, Women attending PMTCT).*<br><br>*In case they test and the husband finds out that the wife is infected he might start questioning her where she got it from, killing themselves or separation. And in case they found themselves both infected, then each will put the blame on the other which in a way affects their relationship (KII 02, Male partner PRIMAL Study).*<br><br>*If the man is not sure of himself he might refuse. If they got it and tested themselves at home and you find that the woman is infected and the man is not infected a fight might breakout and the man might chase away the woman from home. ...men think that women are promiscuous; that they have another sexual partner yet for men; you can even marry another woman when you still have your first wife and it is okay. A man cannot look on when his wife has another partner; he can even decide to kill her. ...if it is the man who has it and the woman is HIV negative, in most cases women are patient, she might get annoyed and choose to stay in another bedroom but she will not leave the home (KII 03, Male Partner PRIMAL Study).*<br><br>***Intentional Transmission of HIV to partner***

	If this person tests him or herself and gets to know that he/she is HIV positive; mark you is alone who knows that is HIV positive, he/she will say "I have also been infected why not infect other people as well! Why should I die alone?" it is very dangerous. You rather have the other testing but not this one of self-testing. I would maybe recommend self-testing for couples but not for everybody (FGD 2, Women attending PMTCT)<br><br>*When you already know that you are infected and you don't want to miss out on that person you have got, you can alter the results. It is easy to lie to someone if she does not know how to use the test kit, whatever you tell her, she might think it is correct (FGD3, male partners of women attending ANC).*<br><br>*Some people may test themselves and if they find they are HIV positive, especially men, they will delay going together to the health facility and the women may get infected in the process (KI – 1 Woman PRIMAL Study).*<br><br>***Failure to enroll in HIV care***<br><br>*I will just call my husband, prick and wait for the results but what will happen after the results. The way forward might not be the best (KII 2- HW PMTCT).*<br><br>*Will people understand the way forward after testing HIV positive? My husband has tested positive and I am negative . . . then what next? Because these people will not be counselled before testing, they will prick and wait for the results but what will happen after the results? They will not go to the hospital for management or they will get divorced depending on the results. (KII 1, Health worker)*

	Self-testing is not good, some people have never attended school and they will never be able to interpret the results. And in case he tested himself he will not encourage himself to go to the clinic and start taking medication. Some people will not even know which hospital to go to for treatment. Some fear going to hospitals near their homes because they do not want to be known. But if you are at a health facility you will be told about other clinics …. You can then determine where you feel you won't find anyone who knows you (KII 2- Male Partner PRIMAL study).<br><br>*When people test themselves, they may not go to enroll in care. Some might fear to go to health centres and fail or delay to start treatment unlike the ones tested at health centres who these days are started on ARVs immediately (FGD 5, HWS providing PMTCT and ANC)*<br><br>*I do not think people would self-test themselves…it would not work because people would not come for other services. Remember when you are tested in the community you are linked to a hospital there and then. But when someone self-tests him or herself she will just remain there. She will remain there if results are negative which will be okay but when she remains there when results are positive then that will not be appropriate. That individual needs to be tested by a health worker and refer for other services (KII- 02 Health worker)*<br><br>*When some people find that they are HIV positive, they may not get the courage to go to the health centre to start ARVs, that may be a big challenge but when you test at a health centre you get help and guidance from health workers and if you have questions, there is someone to answer them (FGD 2, Women attending PMTCT).*<br><br>***Challenges disclosing HIV positive results***<br><br>*I think fear. If one of them is HIV positive and I do not want to disclose my results. …whereas if it*

	*is done at the facility one will test and keep quiet. So that disclosure might be a barrier where one does not want to disclose. I think it comes back to the same as those who come here…fear of a man running away, ... fear to be beaten, abused, domestic violence...if you are a pregnant mother who has tested HIV positive and the man is negative…a fight can spark out (*KII- 5 Counselor*).*<br><br>*Men do not want to test themselves, they fear that they might test together and their partners get to know that they have HIV and everything changes at home. They can even choose to test but still don't tell their partners (KII 03-* Male Partner PRIMAL study*).*<br><br>*Most people will not tell others when they test and find they are HIV positive. They fear to be blamed for being the ones who brought the infection in the family. It takes a lot of encouragement and support from the health worker for disclosure to happen. But this will be missing in self-testing (FGD 2, women attending PMTCT).*<br><br>*Self-testing reason being that confidentiality is high; maybe let me first talk about confidentiality. We are having many issues of confidentiality when it comes to HIV because of stigmatization. But if somebody knows her status alone and is willing to share with another person I think that would be better for the whole community. Because if I know my own secret I won't be able to air it out and when I want I will air it out but if I don't want I can keep it to myself. (FGD 4, HWs PRIMAL study).*<br><br>***Other challenges (high cost, limited availability of kits)***<br>*You see, you have to buy it and in those pharmacies in the villages they may not have them (self-testing kits).*<br>*They might be in big pharmacies and people may not know the name of those things (test kits) and others may not have money and other may not have knowledge about it. Maybe if they can talk*

	about them in the adverts; on TVs, Radios etc but I do not think people will pick that idea (KII 04, Woman PRIMAL study).<br><br>*The high cost of test-kits can hinder use of the testing approach. Unless the kits are given for free like condoms (FGD 1, HIV genitive pregnant women).*<br><br>*Even if people wanted to test themselves, will the test kits be available in all areas especially villages. This will be a challenge (FGD 3, male partners of women attending ANC).*<br><br>*The high cost of testing kits will limit many people from using the approach. You see even if government was to make them (kits) available for free, they will run out and people will need to buy them and when they don't have the money they will not test (FGD 5, HWs providing PMTCT and ANC services).*
Suggestions on integrating HIV self-testing in the PMTCT program	*Community education about HIVST including use of community demonstration on HIV self-testing*<br><br>*Communities should be sensitized about HIV self-testing, how it works and what people should do next. The training should include demonstrating how those kits work (FGD 4, HWs PRIMAL study).*<br><br>*The reason why I would go for self-testing is that, especially the women, can access their husbands who are busy…so if we empower her and give her these strips and really tell her what to do in case the results turn out negative or positive, she can easily link the husband in case he is positive to health workers and seek help. (FGD 4, HWs PRIMAL study).*<br><br>*Those who maybe to the level of primary seven at least know something and if you demonstrate to them and then go talk to their partners and if he agrees they can test from home or they can call us. We can empower the women because it is them who will get in touch with men (FGD 4, HWs PRIMAL study).*

	More sensitization of the public through maybe media…TVs, Radios…through church leaders, LCs and even some community activities. I think through musicians; they can be used to do that… communicating messages through songs. Like they support the cancer people; they schedule cancer runs…maybe if that can be done to promote self-testing and encourage men come on board (KII 05 -Counselor).<br><br>*...men should be found wherever they are and get sensitized... I think, if say we are going to sensitize people, the person who heads that community should be the one to do it and tell men that "Tomorrow all men should come for this or that and whoever doesn't come he will be fined" and I think they will listen (KII 01- Woman PRIMAL study).*<br><br><br>*Provide supportive information, education and communication materials/ sensitization on the use of HIV self-test kits*<br><br>*I think we can go back to health education and tell them that pregnant women are already vulnerable group…so, they are at a higher rate of getting infections in case of…yes they are at a higher rate of getting infections from you. So it is better to test for HIV and self-testing can help (KII- 02 Health worker).*<br><br>*Radio announcements, TV, drama [acting plays]…TV talk shows. Even radio talks where people call in…I think that can also work to increase awareness about self-testing (KII- 05 Counselor).*<br><br>*We need posters in English and in local languages explaining the steps for using HIV self-testing and what to do if one tests HIV positive or HIV negative. I have not seen these posters. It should be like how male circumcision is promoted (FGD 5, HWs providing PMTCT and ANC services).*

	I think men should be used to reach their fellow men to sensitize them on HIV self-testing. ...because they listen to them [each other] more. There are men hardly listen to their women (FGD 4, HWs PRIMAL study).<br><br>*Make test kits accessible at no or low cost*<br><br>*If you want the HIV self-testing to work, take the kits to villages and should be at no cost like condoms are. Some villages do not have pharmacies (FGD 4, HWs PRIMAL Study).*<br><br>*May be if HIV self-testing kits should be given for free at health centres and in communities to make people access them when they need them (FGD, HWs providing PMTCT and ANC services).*<br><br>*Train health workers and community leaders on the benefits and use of HIV self-test kits*<br><br>*Maybe they first counsel those people and then they give the HIV test-kits but just to give [without counselling] is not easy (KII 02, Woman PRIMAL study).*<br><br>*You could first empower them, train them, do thorough training and all that. The advantages and disadvantages can come in but not just coming in but through training them, empowering them, counselling such that whatever results they get or whatever outcome they get they are aware or there is a lot of awareness (FGD 4, HWs PRIMAL study).*<br><br>*There is need to train health workers to educate people because some health workers may just give out test kits like condoms without educating people on their use (KII- 3 Male peer Educator).*<br><br>*We need to intensify our sensitization on HIV self-testing and demonstrate to the men on how to use*

	them. I think it can go along with HIV counselling and demonstrations. (FGD 5, HWs providing PMTCT and ANC services).<br><br>*More sensitization through media should also work well. Recruit health workers with enough knowledge. I think when we involve more male peers they will make a difference. (FGD 5, HWs providing PMTCT and ANC services).*<br><br>*We should also involve local leaders like the LCs because the people listen to them a lot and if they promote it (HIV self-testing) I think it can increase the use. (FGD 4, HWS PRIMAL study).*<br><br>***Other Comments***<br><br>*In my view I feel it is not appropriate because it might cause harm at home. He shouldn't and I would then give him the reasons as to why he shouldn't. You know, finding out positive results is not something simple…For a counsellor to breakout such news of positive results requires a lot of time and you can't say it directly. When some people get to know that they have HIV they can develop pressure and faint. Maybe if someone tests only himself but not his wife or children (KII – 03, Male peer educator).*<br><br>*No we shouldn't. So 9 out of 10 would tell the minister not to promote HIV self-testing (FGD 4, HWs PRIMAL Study).*<br><br>*Self-testing could be a solution because many partners just fear to go in community areas or public areas to test. But initially when a couple is at home or somewhere else I think they can better talk to each other and convince someone into testing (FGD 4, HWS PRIMAL study).*<br><br>*I would not recommend to implement HIV self-testing. Marriages might start breaking up a lot. If*

	you test from a hospital a health worker will be able to tell you that it is good to look after each other and why it is so important. If one is infected, you can still be together so that you are able to see your children grow. I would conclude by telling the minister not to implement it (KII 01-Woman PRIMAL study).<br><br>*We should go ahead and implement HIV self-testing it can help some people (FGD 1, HIV negative pregnant women)*
